# Alpha-Fetoprotein, Protein Induced by Vitamin K Absence or Antagonist II and Glypican-3 for the Detection and Prediction of Hepatocellular Carcinoma in Patients with Cirrhosis of Viral Etiology

**DOI:** 10.3390/cancers12113218

**Published:** 2020-10-31

**Authors:** Gian Paolo Caviglia, Michela Ciruolo, Maria Lorena Abate, Patrizia Carucci, Emanuela Rolle, Chiara Rosso, Antonella Olivero, Giulia Troshina, Alessandra Risso, Aurora Nicolosi, Davide Giuseppe Ribaldone, Angelo Armandi, Francesco Tandoi, Giorgio Maria Saracco, Elisabetta Bugianesi, Alessia Ciancio, Silvia Gaia

**Affiliations:** 1 Department of Medical Sciences, University of Turin, 10100 Turin, Italy; marialorena.abate@unito.it (M.L.A.); chiara.rosso@unito.it (C.R.); antonella.olivero@unito.it (A.O.); yulia.troshina@unito.it (G.T.); aurora.nicolosi999@edu.unito.it (A.N.); davidegiuseppe.ribaldone@unito.it (D.G.R.); angelo.armandi@unito.it (A.A.); giorgiomaria.saracco@unito.it (G.M.S.); elisabetta.bugianesi@unito.it (E.B.); alessia.ciancio@unito.it (A.C.); 2 Division of Gastroenterology, Città della Salute e della Scienza University-Hospital, 10100 Turin, Italy; michela.ciruolo@unito.it (M.C.); pcarucci@cittadellasalute.to.it (P.C.); erolle@cittadellasalute.to.it (E.R.); alrisso@cittadellasalute.to.it (A.R.); 3 Liver Transplant Unit, General Surgery 2U, Department of Surgical Sciences, Città della Salute e della Scienza University-Hospital, 10100 Turin, Italy; ftandoi@cittadellasalute.to.it

**Keywords:** AFP, BCLC, biomarker, GPC-3, HCC, PIVKA-II

## Abstract

**Simple Summary:**

Circulating biomarkers for the early detection and prediction of hepatocellular carcinoma development are an unmet need. In the present study, we observed that serum values of three biomarkers (namely AFP, PIVKA-II and GPC-3) were significantly different between patients with cirrhosis and those with hepatocellular carcinoma; the best accuracy for the detection of tumors was achieved by a combination of AFP + PIVKA-II. However, PIVKA-II resulted as the only biomarker able to identify patients with cirrhosis at increased risk of hepatocellular carcinoma development. The measurement of PIVKA-II in patients with cirrhosis at risk of tumor development may be useful to tailor personalized surveillance strategies and thus to improve patients’ survival.

**Abstract:**

International guidelines recommend the use of ultrasound as a surveillance tool for hepatocellular carcinoma (HCC) in patients with cirrhosis, while the role of serum biomarkers is still debated. We investigated serum alpha-fetoprotein (AFP), protein induced by vitamin K absence or antagonist II (PIVKA-II) and glypican-3 (GPC-3) diagnostic accuracy for HCC detection and prediction in patients with liver cirrhosis of viral etiology under surveillance. A total of 349 patients (200 cirrhosis and 149 HCC) were enrolled. The 200 patients with cirrhosis consisted of 114 patients still HCC-free after 36 months of follow-up and 86 patients that developed HCC after 13.8 (11.0–19.8) months. AFP, PIVKA-II and GPC-3 were measured in serum samples collected at tumor diagnosis in the 149 patients with HCC, and at the beginning of follow-up in the 200 patients with cirrhosis. The higher performance for HCC detection was observed for PIVKA-II (area under the curve (AUC) = 0.790), followed by AFP (AUC = 0.737) and GPC-3 (AUC = 0.637); the combination of AFP + PIVKA-II improved the diagnostic accuracy to AUC = 0.822. Serum PIVKA-II values, but not AFP and GPC-3, were significantly higher in the 86 cirrhotics that developed HCC compared with the 114 cirrhotics still HCC-free after 36 months of follow-up (*p* = 0.020). PIVKA-II ≥ 55 mAU/mL allowed to identify patients with cirrhosis at higher risk of HCC development (Log-rank test, *p* < 0.001; adjusted Hazard Ratio = 1.99, *p* = 0.001). In conclusion, the measurement of PIVKA-II in patients with cirrhosis may be useful to tailor personalized surveillance strategies.

## 1. Introduction

Liver cancer is the sixth most common cancer worldwide, with 841,000 new cases and 782,000 deaths per year [1]. Hepatocellular carcinoma (HCC) constitutes more than 90% of primary liver cancers, and cirrhosis related to chronic hepatitis B virus (HBV) and hepatitis C virus (HCV) infection represents the key risk factor for the development of tumors [2]. The prognosis of HCC varies greatly according to tumor stage at the time of diagnosis; therefore, a timely detection is crucial in order to allow curative approaches and thus improve patients’ survival [3,4].

Currently, surveillance programs for HCC detection in high risk populations are mainly based on abdominal ultrasonography (US), while the use of serum biomarkers is still a matter of debate [5,6,7]. US is a non-invasive and cost-effective method for screening purposes; the main limitation is represented by its sub-optimal sensitivity, especially for early-stage HCCs [8]. In addition, the presence of a coarse liver echo pattern, obesity, meteorism, chest wall deformities, previous abdominal surgery, scarce patient cooperation and the expertise of the operator are some of the conditions that can affect the US examination [9]. Thus, there is a need to adopt other non-invasive methods in association with US to improve the identification of potential neoplastic lesions.

Among non-invasive biomarkers, alpha-fetoprotein (AFP) has been the most widely investigated in HCC; a recent meta-analysis showed a diagnostic accuracy for HCC detection of 0.767 (0.732–0.803) [10]. However, in the surveillance setting, data on the effectiveness of AFP in combination with US are still conflicting [11,12]. Protein induced by vitamin K absence or antagonist-II (PIVKA-II) is an abnormal prothrombin generated during hepatocytes malignant transformation due to an impaired post-translational carboxylation that showed better diagnostic performance than AFP for the detection of HCC [13]. Furthermore, the combined use of AFP and PIVKA-II showed an improved diagnostic accuracy compared to each single biomarker used alone [10,14]. Remarkably, Saitta and colleagues recently showed that the combination of AFP + PIVKA-II was able to discriminate between neoplastic and nonneoplastic lesions in cirrhotic livers [15]. Serum glypican-3 (GPC-3) is an oncofetal protein which is normally not expressed in adult liver [16]; several studies showed a good performance of GPC-3 for the discrimination between patients with HCC and liver cirrhosis [17], however, data on Caucasian patients are scanty.

Our aim was to investigate AFP, PIVKA-II and GPC-3 diagnostic accuracy for the detection and the prediction of HCC development in patients with cirrhosis of viral etiology under surveillance.

## 2. Results

**Figure 1:**
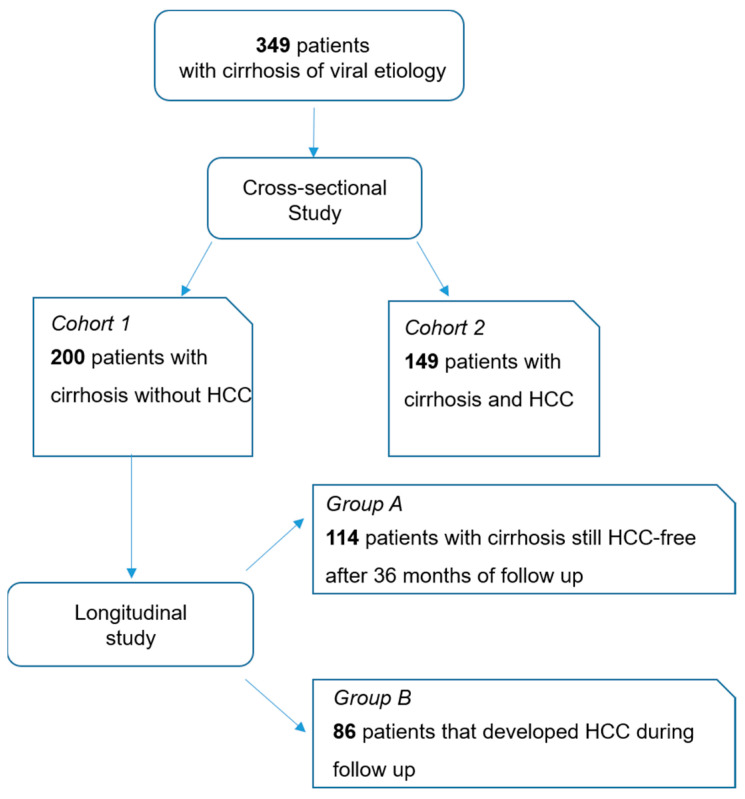
Flow chart of the study. Abbreviations—hepatocellular carcinoma (HCC).

A total of 349 patients (149 patients with HCC and 200 patients with cirrhosis without HCC) were included in the study (Figure 1). The demographic, biochemical and clinical characteristics are reported in Table 1. Overall, most patients were Caucasian (344 out of 349; 98.6%). The 149 patients with HCC were significantly older than the 200 patients with cirrhosis (67, range 31–89 years vs. 61, range 33–82 years; *p* < 0.001). The ratio of males to total patients was 83% (123/149) in patients with HCC and 67% (13/200) in those with cirrhosis (*p* = 0.001). Patients with HCC showed higher alanine aminotransferase (ALT) (*p* = 0.023), higher aspartate aminotransferase (AST) (*p* < 0.001), lower platelet count (*p* < 0.001), lower albumin (*p* < 0.001) and higher total bilirubin (*p* = 0.018) compared to patients with cirrhosis. The majority of patients with HCC had a diagnosis of early tumor (Barcelona Clinic Liver Cancer (BCLC) 0 and A: 77%); 30 patients (20%) had a diagnosis of intermediate stage of HCC (BCLC B) and 4 patients (3%) had a diagnosis of advanced stage tumor.

**Figure 2:**
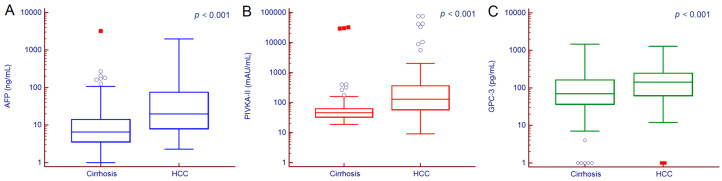
AFP (**A**), PIVKA-II (**B**) and GPC-3 (**C**) serum values in patients with or without HCC. Hollow circles indicate values that are smaller than the lower quartile minus 1.5 times the interquartile range, or larger than the upper quartile plus 1.5 times the interquartile range; red squares indicate values that are smaller than the lower quartile minus 3 times the interquartile range, or larger than the upper quartile plus 3 times the interquartile range. *p* values were calculated by Man–Whitney test. Abbreviations—alpha-fetoprotein (AFP), glypican-3 (GPC-3), hepatocellular carcinoma (HCC), protein induced by vitamin K absence or antagonist II (PIVKA-II).

Median serum values of AFP, PIVKA-II and GPC-3 were significantly different between cirrhotic patients with or without HCC (Figure 2). Serum AFP values were 6.6 (3.6–14.3) ng/mL in patients with cirrhosis and 19.8 (8.1–74.9) ng/mL in those with HCC (*p* < 0.001); PIVKA-II values were 46 (33–63) mAU/mL in patients with cirrhosis and 129 (59–361) mAU/mL in those with HCC (*p* < 0.001); GPC-3 values were 71 (37–163) pg/mL in patients with cirrhosis and 144 (62–246) pg/mL in patients with HCC (*p* < 0.001).

**Figure 3:**
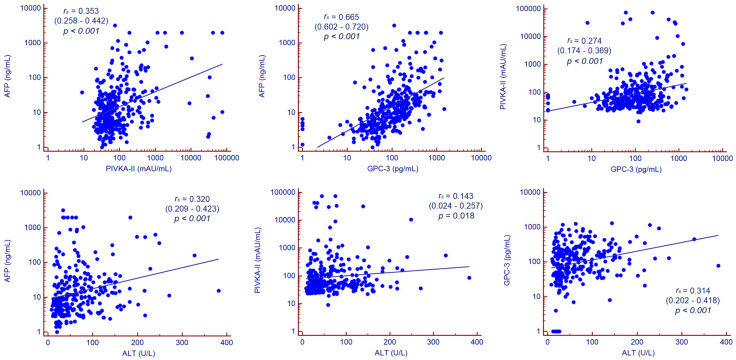
Correlation analysis. Correlation between continuous variables was assessed by Spearman’s rank correlation coefficient. Abbreviations—alpha-fetoprotein (AFP), alanine aminotransferase (ALT), glypican-3 (GPC-3), protein induced by vitamin K absence or antagonist II (PIVKA-II).

Serum values of PIVKA-II were moderately correlated to serum values of AFP and GPC-3, while AFP and GPC-3 showed a strong correlation (Figure 3).

AFP was positively correlated with ALT (*r_s_* = 0.316, 95% CI 0.207–0.417, *p* < 0.001), AST (*r_s_* = 0.396, 95% CI 0.291–0.491, *p* < 0.001), total bilirubin (*r_s_* = 0.156, 95% CI 0.035–0.272, *p* = 0.012) and the size of the major HCC nodule (*r_s_* = 0.289, 95% CI 0.119–0.443, *p* < 0.001) while negatively correlated to platelet count (*r_s_* = −0.238, 95% CI −0.341–−0.129, *p* < 0.001) and albumin (*r_s_* = −0.373, 95% CI −0.479–−0.256, *p* < 0.001).

PIVKA-II was positively correlated to ALT (*r_s_* = 0.143, 95% CI 0.027–0.256, *p* = 0.016), AST (*r_s_* = 0.185, 95% CI 0.069–0.297, *p* = 0.002), total bilirubin (*r_s_* = 0.151, 95% CI 0.030–0.268, *p* = 0.015), HCC nodules number (*r_s_* = 0.228, 95% CI 0.056–0.387, *p* = 0.010) and size of the major HCC nodule (*r_s_* = 0.418, 95% CI 0.262–0.552, *p* < 0.001). 

**Figure 4:**
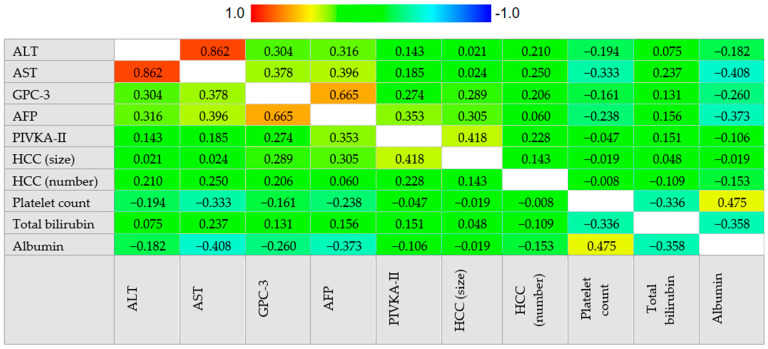
Correlogram. Correlation between continuous variables was assessed by Spearman’s rank correlation coefficient. The cells are colored according to the magnitude of the correlations, ranging from dark red for positive correlations to dark blue for negative correlations. Abbreviations—alpha-fetoprotein (AFP), alanine aminotransferase (ALT), aspartate aminotransferase (AST), glypican-3 (GPC-3), hepatocellular carcinoma (HCC), protein induced by vitamin K absence or antagonist II (PIVKA-II).

GPC-3 was positively correlated to ALT (*r_s_* = 0.304, 95% CI 0.194–0.407, *p* < 0.001), AST (*r_s_* = 0.378, 95% CI 0.271–0.476, *p* < 0.001), HCC nodules number (*r_s_* = 0.206, 95% CI 0.032–0.369, *p* = 0.021) and the size of the major HCC nodule (*r_s_* = 0.289, 95% CI 0.119–0.443, *p* = 0.001) while negatively correlated to platelet count (*r_s_* = −0.161, 95% CI −0.269–−0.049, *p* = 0.005) and albumin (*r_s_* = −0.260, 95% CI −0.377–−0.135, *p* < 0.001). Correlation analysis is summarized in Figure 4.

**Figure 5:**
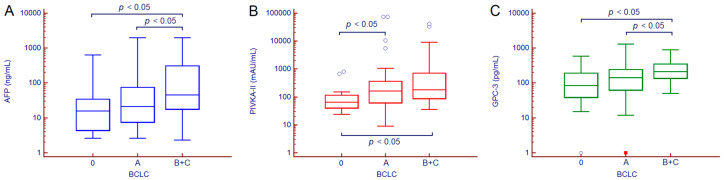
AFP (**A**), PIVKA-II (**B**) and GPC-3 (**C**) serum values in patients with HCC according to BCLC stage. Hollow circles indicate values that are smaller than the lower quartile minus 1.5 times the interquartile range, or larger than the upper quartile plus 1.5 times the interquartile range; red squares indicate values that are smaller than the lower quartile minus 3 times the interquartile range, or larger than the upper quartile plus 3 times the interquartile range. *p* values were calculated by Mann–Whitney test. Abbreviations—alpha-fetoprotein (AFP), Barcelona Clinic Liver Cancer (BCLC), glypican-3 (GPC-3), protein induced by vitamin K absence or antagonist II (PIVKA-II).

Serum values of AFP, PIVKA-II and GPC-3 stepwise increased according to HCC staging (Table 2). By paired comparison, AFP and GPC-3 values were significantly different between patients with very early HCC vs. those with intermediate/advanced HCC and between patients with early HCC vs. those with intermediate/advanced HCC; serum PIVKA-II concentration was significantly different between patients with very early HCC vs. those with early HCC and between patients with very early HCC vs. those with intermediate/advanced HCC (Figure 5).

### 2.1. Diagnostic Accuracy of AFP, PIVKA-II and GPC-3 for the Detection of HCC

**Figure 6:**
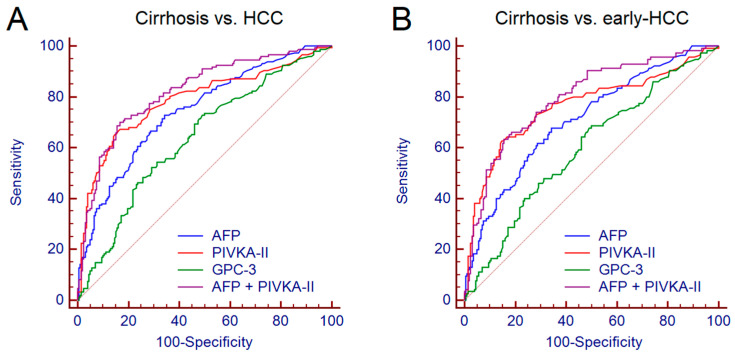
Comparison between the performance of AFP, PIVKA-II, GPC-3 and AFP + PIVKA-II for the discrimination between patients with cirrhosis and those with HCC (**A**) and between patients with cirrhosis and patients with early HCC (**B**). AUC was assessed by receiver operating characteristic curve analysis. Abbreviations—alpha-fetoprotein (AFP), area under the curve (AUC), glypican-3 (GPC-3), hepatocellular carcinoma (HCC), protein induced by vitamin K absence or antagonist II (PIVKA-II).

The diagnostic accuracy of AFP, PIVKA-II and GPC-3 for the discrimination between patients with cirrhosis and those with HCC was assessed by receiver operating characteristic (ROC) curve analysis. The values of area under the curve (AUC), sensitivity (Se), specificity (Sp), positive likelihood ratio (+LR) and negative likelihood ratio (−LR) are reported in Table 3. PIVKA-II showed the higher performance with AUC = 0.790, followed by AFP (AUC = 0.737) and by GPC-3 (AUC = 0.637). The best performance derived from the combination of AFP + PIVKA-II (AUC = 0.822) (Figure 6A). No further improvement was observed following the addition of GPC-3 to AFP + PIVKA-II.

Afterwards, we assessed the diagnostic accuracy of the biomarkers for the discrimination between patients with cirrhosis and patients with early HCC (BCLC 0 and A, *n* = 115). Overall, we observed only a moderate reduction of performance (Table S1); the combination of AFP + PIVKA-II maintained a good accuracy, showing AUC = 0.802 (Figure 6B). 

Since the two groups of patients (i.e., patients with cirrhosis and patients with HCC) showed significant differences regarding demographic and biochemical features, we performed a multivariate logistic regression analysis to test whether the observed differences may impact on the performance of AFP, PIVKA-II and GPC-3 (Table 4). We observed that AFP and PIVKA-II, but not GPC-3, resulted significantly and independently associated with the diagnosis of HCC.

### 2.2. Prediction of HCC Development in Patients with Cirrhosis under Surveillance

The group of 200 patients with cirrhosis, HCC-free at the time of blood sampling and biomarkers testing, consisted of 114 patients still HCC-free after 36 months of follow-up and 86 patients that developed HCC after a median follow-up of 13.8 (11.0–19.8) months. The characteristics of the two subgroups of patients are reported in Table 5. Patients with cirrhosis that developed HCC during the follow-up were older than patients that did not develop the tumor (65, range 35–82 years vs. 57, range 33–82 years; *p* < 0.001) and there was a trend towards a higher male prevalence in the former group compared to the latter (70/114 vs. 64/86; *p* = 0.068). Regarding biochemical features, patients that developed HCC had a lower platelet count compared to patients still HCC-free after 36 months of follow-up (*p* < 0.001). At baseline, no differences in AFP and GPC-3 serum levels were observed between patients that developed HCC or not (*p* = 0.851 and *p* = 0.844, respectively). Conversely, PIVKA-II serum values were significantly higher in patients that developed HCC compared to those still HCC-free after 36 months of follow-up (Figure S1). 

**Figure 7:**
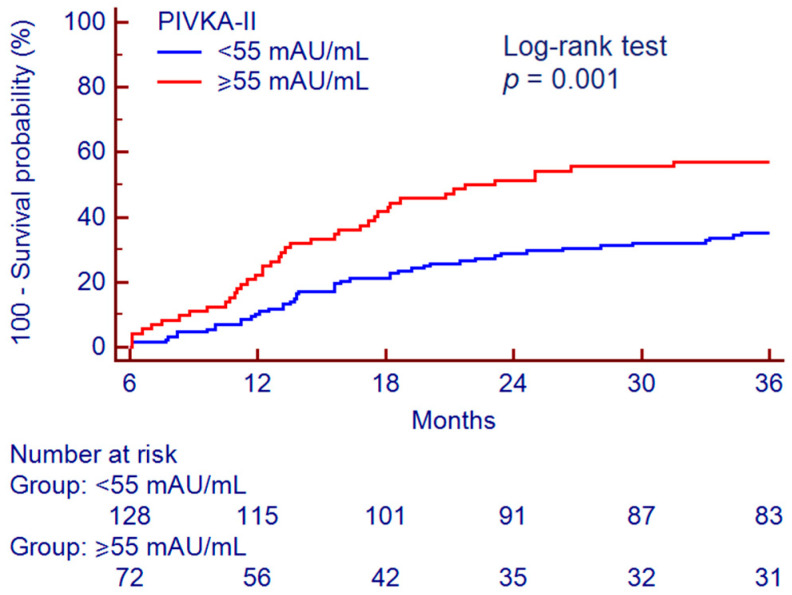
Survival curve for the prediction of HCC development according to PIVKA-II. Survival curve analysis was performed according to the Kaplan–Meier method; the difference between the curves was assessed by Log-rank test. Abbreviations—protein induced by vitamin K absence or antagonist II (PIVKA-II).

By a Youden *J* statistic, we identified a PIVKA-II cut-off value of 55 mAU/mL for data dichotomization and subsequent analysis. Kaplan–Meier analysis showed different survival curves (Figure 7); among the 128 patients with baseline serum PIVKA-II < 55 mAU/mL, 45 (35.2%) developed HCC, while among the 72 patients with baseline serum PIVKA-II ≥ 55 mAU/mL, 41 (56.9%) developed the tumor during the 36 months follow-up (*p* = 0.001).

At multivariate Cox proportional hazards regression analysis corrected for age and platelet count, PIVKA-II ≥ 55 mAU/mL resulted in a significant independent factor associated to HCC development (hazard ratio (HR) = 1.99, 95% CI 1.25–3.19, *p* = 0.004) (Table 6).

## 3. Discussion

The early detection of HCC, or even its prediction, represents the major goal in order to improve patients’ survival; thus, the identification of biomarkers able to reflect molecular alterations preceding tumor development is an unmet need. In the present study, we observed that serum values of AFP, PIVKA-II and GPC-3 were significantly different between patients with cirrhosis and those with HCC; however, the best accuracy for the detection of tumors was achieved by the combination of AFP + PIVKA-II. Furthermore, PIVKA-II resulted to be the only biomarker able to identify patients with cirrhosis at increased risk of HCC development.

To date, several candidate biomarkers have been investigated in order to improve the detection of HCC in patients with cirrhosis under surveillance [16,17]. Among novel biomarkers, non-coding circulating RNAs such as microRNAs, showed interesting results [18]; however, their main limitation is represented by the absence of a standardized method for their quantitation in body fluids [19]. On the other hand, traditional biomarkers such as AFP have been intensely debated due to their suboptimal accuracy. Nonetheless, recent technical advancements in the analytical methods led to an improvement of their performance [20]. In addition, different studies suggested that the combination of different classes of biomarkers and the use of scores based on different clinical or demographic features further improved early HCC detection rates [21,22,23,24,25]. In our study, in agreement with previous findings [10,26,27,28], the combined use of AFP + PIVKA-II led to a good diagnostic accuracy for the detection of HCC (AUC = 0.822); even including in the analysis only patients with early HCC, the combined performance of AFP + PIVKA-II remained satisfactory (AUC = 0.802). On the other hand, GPC-3 showed only a moderate diagnostic accuracy. Previous reports showed contradictory results concerning the accuracy of GPC-3 for the detection of HCC [17,29,30,31]; as a matter of fact, the control population included in the studies (i.e., patients with cirrhosis or chronic liver disease or healthy subjects), the overall clinical and biochemical characteristics of the patients enrolled, the different assays used for the measurement of GPC-3 may have accounted for these conflicting findings. 

Although the combined used of AFP + PIVKA-II showed a good performance for the detection of HCC, the added value of a non-invasive biomarker is represented by the ability to predict tumor development before imaging discovery. Recently, Loglio and colleagues investigated the performance of PIVKA-II alone or in combination with AFP in 212 nucleos(t)ide analogues-treated patients with HBV-related cirrhosis (64 HCC and 148 HCC-free controls) for the early detection of HCC during surveillance [32]. Interestingly, serum PIVKA-II values raised above the identified cut-off (82 mAU/mL) in 53% of the patients already 6–18 months before HCC detection, showing an accuracy of 86% (Se = 54% and Sp = 100%); combining PIVKA-II with AFP, the accuracy further improved to 90% (Se = 67% and Sp = 100%). Similarly, a real-world study carried out in China showed that the measurement of serum PIVKA-II in patients with HBV-related cirrhosis allowed to stratify the patients according to the cumulative incidence of HCC in a two-years follow up [33]; HCC developed in the 82.0% of patients with elevated PIVKA-II values compared to the 54.1% of patients with low PIVKA-II (*p* < 0.001). Another recent study by Ricco and colleagues investigated the time-related variability of AFP and PIVKA-II in serum samples of 418 patients with cirrhosis (124 HCC and 294 HCC-free controls) predominantly of viral etiology undergoing standard HCC surveillance by US [34]. It has been observed that both AFP and PIVKA-II increased over time in patients that developed HCC, but among HCC-free controls the time-related changes of PIVKA-II were more stable than AFP; a 5% increase of Log_10_ PIVKA-II serum values resulted significantly for the prediction of HCC development. Consistently, we found that serum PIVKA-II levels were significantly different between patients with cirrhosis that developed HCC during 36 months follow-up from those still HCC-free at the end of the observation period. In our series, PIVKA-II, with a cut-off value set at 55 mAU/mL, was able to early identify patients at higher risk of HCC development (HR = 1.99); indeed, the incidence of HCC during the observation period was almost double in patients with baseline serum PIVKA-II ≥ 55 mAU/mL. However, the issue concerning the optimal cut-off for PIVKA-II remains unsolved and the heterogeneity among the different studies is not negligible. The most likely explanation for this discrepancy could lie in the diverse ethnicity, clinical characteristics (such as cirrhosis prevalence, liver disease activity, HCC stage) and any underlying liver disease etiology of the patients enrolled in the different studies; last but not least, the analytical method used for biomarker determination may have impacted on the obtained results. 

### Study Limitations

The major limitations of our study are represented by the retrospective design and the lack of a cost-effectiveness analysis. Consequently, we were not able to evaluate the benefits deriving from the combination of US and serum biomarkers for HCC surveillance in terms of improved accuracy, better survival and increased quality-adjusted life years. Furthermore, there were several differences between cases and controls concerning demographic and biochemical features. Despite it being well known that older age, male gender and disease activity are important risks factors associated to a higher likelihood of developing HCC, our analysis was adjusted for these potential confounders. Thus, we believe that the results are reasonably robust and provide further evidences concerning the usefulness of PIVKA-II in the setting of HCC surveillance. Further prospective studies including consecutive patients with cirrhosis under surveillance for the risk of HCC development are needed to concretely prove the usefulness of PIVKA-II measurement in the real-life clinical setting.

## 4. Materials and Methods

### 4.1. Patients

This retrospective study included patients with cirrhosis of viral etiology with and without a new diagnosis of HCC, recruited at the outpatient clinic of the Unit of Gastroenterology of Città della Salute e della Scienza di Torino—Molinette Hospital, Turin, Italy, between November 2012 and January 2018. 

For all patients, the inclusion criteria were age ≥18 years, serum hepatitis B surface antigen-positivity or anti-HCV-positivity, diagnosis of cirrhosis, availability of a serum sample at the time of HCC diagnosis and signed written informed consent. In addition, for patients with cirrhosis under surveillance, a minimum of 3-year US follow-up after the collection of serum sample was required. Patients in anticoagulant therapy were excluded.

The presence of cirrhosis was determined by liver elastography (FibroScan^®^, Echosens™, Paris, France) or by hepatic ultrasound features and endoscopic signs of portal hypertension [35,36]. The diagnosis of HCC was achieved by histological examination or by contrast-enhanced imaging methods showing the radiological hallmark of HCC (i.e., the combination of hypervascularity in late arterial phase and washout on portal venous and/or delayed phases), following international guidelines [5]. HCC was classified according to the BCLC staging system (0 = very early; A = early; B = intermediate; C = advanced; D = end-stage) [5]. 

Serum samples were collected in polypropylene 2 mL tubes labelled with the study participant identification code and stored at −80 °C until analysis. Study procedures were compliant with the principles of the Declaration of Helsinki. All patients gave their written informed consent and the study was approved by the Institutional Ethics Committee (CEI-452). 

### 4.2. Measurement of Serum AFP, PIVKA-II and GPC-3

Serum levels of AFP and PIVKA-II were determined on the fully automated chemiluminescent enzyme immunoassay (CLEIA) system Lumipulse^®^ G600 II (Fujirebio Inc., Tokyo, Japan) using Lumipulse^®^ G AFP-N and Lumipulse^®^ G PIVKA-II reaction cartridges according to manufacturer’s instructions. AFP concentrations were given in ng/mL, while PIVKA-II values were reported in mAU/mL [37]. Detection limit of AFP and PIVKA-II assays were 0.075 ng/mL and 1.37 mAU/mL, respectively.

Serum levels of GPC-3 were measured by enzyme-linked immunosorbent assay (ELISA) (CanAg Glypican-3 EIA, Fujirebio Diagnostics AB, Gothenburg, Sweden) according to thes manufacturer’s instructions. Serum values of GPC-3 were reported in pg/mL. 

### 4.3. Statistical Analysis

Continuous variables were expressed as median and interquartile ranges (IQR). D’Agostino-Pearson test was used to test data normality. Mann–Whitney test and Fisher’s exact test were used to compare continuous and categorical variables, respectively. Kruskal–Wallis test was used to compare continuous variables among more than two independent groups. Correlation between continuous variables was assessed by Spearman’s rank correlation coefficient (*r_s_*). To evaluate diagnostic performance of AFP, PIVKA-II and GPC-3 alone or in combination, AUC values, Se, Sp, +LR and −LR were assessed by ROC curves analysis. Multivariate logistic regression analysis was performed to evaluate whether selected variables were significantly and independently associated with HCC (dependent variable). The comparison between survival curves was performed by the Kaplan–Meier method; the difference between the curves was evaluated by Log-rank test. Multivariate Cox proportional-hazards regression was performed to calculate the Hazard Ratios (HR) for HCC development.

A two-tailed *p* < 0.05 was considered statistically significant. All statistical analyses were performed using MedCalc software, version 18.9.1 (MedCalc bvba, Ostend, Belgium). 

## 5. Conclusions

In conclusion, PIVKA-II showed a higher diagnostic accuracy than AFP and GCP-3 for the discrimination between patients with or without HCC; furthermore, the combination of AFP + PIVKA-II was superior to PIVKA-II alone for HCC detection. However, the possibility to early identify patients at higher risk of HCC development prior to tumor detection is crucial in order to tailor personalized surveillance strategies and thus to improve survival. The evidences provided by this study suggest that the measurement of serum PIVKA-II may allow to stratify patients with cirrhosis under surveillance according to the likelihood of tumor development and may be useful to select patients that could benefit from a closer monitoring.

## Figures and Tables

**Table 1:** Characteristics of the patients included in the study according to the diagnosis of hepatocellular carcinoma.

Characteristics	Cirrhosis	HCC	*p* Value
Patients, *n*	200	149	
Age (years), median (range)	61 (33–82)	67 (31–89)	<0.001
Male gender, *n* (%)	134 (67%)	123 (83%)	0.001
Caucasian ethnicity, *n* (%)	197 (99%)	147 (99%)	1.000
Etiology			
HBV, *n* (%)	52 (26%)	40 (27%)	0.902
HCV, *n* (%)	148 (74%)	109 (73%)	
ALT (U/L), median (IQR)	45 (26–86)	63 (34–104)	0.023
AST (U/L), median (IQR)	45 (28–80)	77 (41–115)	<0.001
Platelets (×10^9^/L), median (IQR)	123 (79–177)	101 (66–129)	<0.001
Albumin (g/dL), median (IQR)	4.1 (3.7–4.4)	3.9 (3.4–4.1)	<0.001
INR, median (IQR)	1.11 (1.02–1.20)	1.15 (1.06–1.25)	0.103
Total Bilirubin (mg/dL), median (IQR)	0.9 (0.7–1.2)	1.0 (0.7–1.6)	0.018
Child-Pugh Score			
A, *n* (%)	188 (94%)	131 (88%)	0.034
B, *n* (%)	12 (6%)	18 (12%)	
BCLC Score			
0, *n* (%)		33 (22%)	
A, *n* (%)		82 (55%)	
B, *n* (%)		30 (20%)	
C, *n* (%)		4 (3%)	
HCC nodules			
1, *n* (%)		87 (58%)	
2, *n* (%)		34 (23%)	
3, *n* (%)		22 (15%)	
>3, *n* (%)		6 (4%)	
Size of major nodule (mm), median (IQR)		22 (17–33)	

*p* values for quantitative variables were calculated by Mann–Whitney test while *p* values for categorical variables were calculated by Fishers’ Exact test. Abbreviations—alanine aminotransferase (ALT), aspartate aminotransferase (AST), Barcelona Clinic Liver Cancer (BCLC), hepatitis B virus (HBV), hepatocellular carcinoma (HCC), hepatitis C virus (HCV), international normalized ratio (INR), interquartile range (IQR), number (*n*).

**Table 2:** AFP, PIVKA-II and GPC-3 serum values in patients with HCC according to BCLC stage.

		BCLC Staging		
Biomarkers	0	A	B/C	*p* Value
Patients, *n*	33	82	34	
AFP (ng/mL), median (IQR)	15.9 (4.4–34.4)	21.4 (7.5–74.7)	45.2 (17.8–367.1)	0.004
PIVKA-II (mAU/mL), median (IQR)	66 (40–115)	162 (62–366)	180 (87–742)	<0.001
GPC-3 (pg/mL), median (IQR)	85 (39–194)	141 (62–243)	213 (138–352)	<0.001

*p* values were calculated by Kruskal–Wallis test. Abbreviations—alpha-fetoprotein (AFP), Barcelona Clinic Liver Cancer (BCLC), glypican-3 (GPC-3), interquartile range (IQR), protein induced by vitamin K absence or antagonist II (PIVKA-II).

**Table 3:** Diagnostic accuracy of AFP, PIVKA-II, GPC-3 alone or in combination for the discrimination between patients with cirrhosis and those with HCC.

Biomarkers	AUC (95% CI)	Cut-off **	Se	Sp	+LR	-LR
AFP	0.737 (0.688–0.783)	>9.7 ng/mL	72%	66%	2.11	0.43
PIVKA-II	0.790 (0.744–0.832)	>73 mAU/mL	68%	84%	4.11	0.39
GPC-3	0.637 (0.584–0.688)	>73 pg/mL	73%	51%	1.49	0.53
AFP + PIVKA-II *	0.822 (0.777–0.860)	>0.41	70%	84%	4.23	0.36

The biomarkers combination was carried out by logistic regression analysis. * The obtained formula of the model is: y = −5.09676 + 1.19356*Log AFP + 1.81397*Log PIVKA-II; the probability (*p*) of HCC is given by: *p* = 1/(1 + e^−y^). ** Identified by Youden *J* statistic. AUC values were calculated by receiver operating characteristic curve analysis. Abbreviations—alpha-fetoprotein (AFP), area under the curve (AUC), glypican-3 (GPC-3), hepatocellular carcinoma (HCC), protein induced by vitamin K absence or antagonist II (PIVKA-II), sensitivity (Se), specificity (Sp), positive likelihood ratio (+LR), negative likelihood ratio (−LR).

**Table 4:** Multivariate analysis for factors associated to HCC.

Variables *	OR (95% CI)	*p* Value
Age (years)	1.07 (1.03–1.10)	<0.001
Male gender	2.46 (1.12–5.34)	0.023
ALT (U/L)	0.99 (0.98–1.01)	0.926
AST (U/L)	1.00 (0.99–1.01)	0.603
Platelets (×10^9^/L)	0.99 (0.98–0.99)	0.044
Child-Pugh score B	2.43 (0.77–7.79)	0.136
AFP > 9.7 ng/mL	2.42 (1.12–5.26)	0.025
PIVKA-II > 73 mAU/mL	8.57 (4.38–16.74)	<0.001
GPC-3 > 73 pg/mL	0.72 (0.31–1.66)	0.438

* Albumin and Total bilirubin were not added in the analysis since it already included in Child-Pugh score. *p* values were calculated by multiple stepwise logistic regression analysis. Abbreviations—alpha-fetoprotein (AFP), alanine aminotransferase (ALT), aspartate aminotransferase (AST), confidence interval (CI), glypican-3 (GPC-3), hepatocellular carcinoma (HCC), odds ratio (OR), protein induced by vitamin K absence or antagonist II (PIVKA-II).

**Table 5:** Characteristics of the patients with cirrhosis that developed or not HCC during 36 months of follow-up.

Characteristics	No HCC Development *	HCC Development **	*p* Value
Patients, *n*	114	86	
Age (years), median (range)	57 (33–82)	65 (35–82)	<0.001
Male gender, *n* (%)	70 (61%)	64 (74%)	0.068
Caucasian ethnicity, *n* (%)	113 (99%)	84 (98%)	0.578
Etiology			
HBV, *n* (%)	28 (25%)	25 (29%)	0.519
HCV, *n* (%)	86 (75%)	61 (71%)	
ALT (U/L), median (IQR)	55 (28–98)	42 (24–72)	0.096
AST (U/L), median (IQR)	51 (28–80)	51 (29–82)	0.761
Platelets (×10^9^/L), median (IQR)	142 (92–190)	88 (61–143)	<0.001
Albumin (g/dL), median (IQR)	4.2 (3.8–4.4)	4.0 (3.6–4.3)	0.124
INR, median (IQR)	1.10 (1.02–1.16)	1.15 (1.06–1.25)	0.075
Total Bilirubin (mg/dL), median (IQR)	0.9 (0.7–1.2)	0.9 (0.7–1.2)	0.932
Child-Pugh Score			
A, *n* (%)	109 (96%)	80 (93%)	0.535
B, *n* (%)	5 (4%)	6 (7%)	
AFP (ng/mL), median (IQR)	7.0 (4.0–13.1)	6.0 (3.2–19.1)	0.851
PIVKA-II (mAU/mL), median (IQR)	43 (32–57)	52 (35–73)	0.020
GPC-3 (pg/mL), median (IQR)	69 (37–170)	80 (37–152)	0.844

* Patients with cirrhosis HCC-free during 36 months follow-up. ** Patients with cirrhosis that developed HCC within 36 months of follow-up. *p* values for quantitative variables were calculated by Mann–Whitney test while *p* values for categorical variables were calculated by Fishers’ Exact test. Abbreviations—alanine aminotransferase (ALT), aspartate aminotransferase (AST), Barcelona Clinic Liver Cancer (BCLC), hepatitis B virus (HBV), hepatocellular carcinoma (HCC), hepatitis C virus (HCV), international normalized ratio (INR), interquartile range (IQR), number (*n*).

**Table 6:** Multivariate analysis for factors associated to HCC development.

Variables	HR (95% CI)	*p* Value
Age (years)	1.05 (1.03–1.08)	<0.001
Platelets (×10^9^/L)	0.99 (0.99–0.99)	<0.001
PIVKA-II > 55 mAU/mL	1.99 (1.25–3.19)	0.004

*p* values were calculated by multivariate Cox proportional-hazards regression analysis. Abbreviations—confidence interval (CI), hazard ratio (HR), protein induced by vitamin K absence or antagonist II (PIVKA-II).

## Supplementary Materials

The following are available online at https://www.mdpi.com/2072-6694/12/11/3218/s1, Table S1: Diagnostic accuracy of AFP, PIVKA-II, GPC-3 and AFP + PIVKA-II for the discrimination between patients with cirrhosis and those with HCC, Figure S1: AFP (**A**), PIVKA-II (**B**) and GPC-3 (**C**) serum values in patients with cirrhosis that developed or not HCC during 36 months of follow-up.
