# Supplementary material for: Alpha-Fetoprotein, Protein Induced by Vitamin K Absence or Antagonist II and Glypican-3 for the Detection and Prediction of Hepatocellular Carcinoma in Patients with Cirrhosis of Viral Etiology
Source: Cancers (Basel). 2020 Oct 31;12(11):3218. doi: 10.3390/cancers12113218 (PMC7692611; doi:10.3390/cancers12113218)
Supplement: Supplementary file 1 [file cancers-12-03218-s001.pdf]

cancers

Supplementary material

Alpha-fetoprotein, Protein Induced by Vitamin K Absence or Antagonist II and Glypican-3 for the Detection and Prediction of Hepatocellular Carcinoma in Patients with Cirrhosis of Viral Etiology

Gian Paolo Caviglia, Michela Ciruolo, Maria Lorena Abate, Patrizia Carucci, Emanuela Rolle, Chiara Rosso, Antonella Olivero, Giulia Troshina, Alessandra Risso, Aurora Nicolosi, Davide Giuseppe Ribaldone, Angelo Armandi, Francesco Tandoi, Giorgio Maria Saracco, Elisabetta Bugianesi, Alessia Ciancio and Silvia Gaia

Table S1. Diagnostic accuracy of AFP, PIVKA-II, GPC-3 and AFP + PIVKA-II for the discrimination between patients with cirrhosis and those with HCC.

Biomarkers	AUC (95% CI)	Cut-off	Se	Sp	+LR	-LR
AFP	0.708 (0.654–0.757)	>9.7 ng/mL	67%	66%	1.98	0.50
PIVKA-II	0.766 (0.715–0.811)	>73 mAU/mL	65%	84%	3.92	0.42
GPC-3	0.597 (0.540–0.652)	>73 pg/mL	68%	51%	1.38	0.63
AFP + PIVKA-II	0.802 (0.753–0.844)	>0.41	59%	86%	4.19	0.48

The combination of two or more biomarkers was performed by logistic regression analysis. Abbreviations–alpha-fetoprotein (AFP), glypican-3 (GPC-3), hepatocellular carcinoma (HCC), protein induced by vitamin K absence or antagonist II (PIVKA-II), sensitivity (Se), specificity (Sp), positive likelihood ratio (+LR), negative likelihood ratio (−LR).

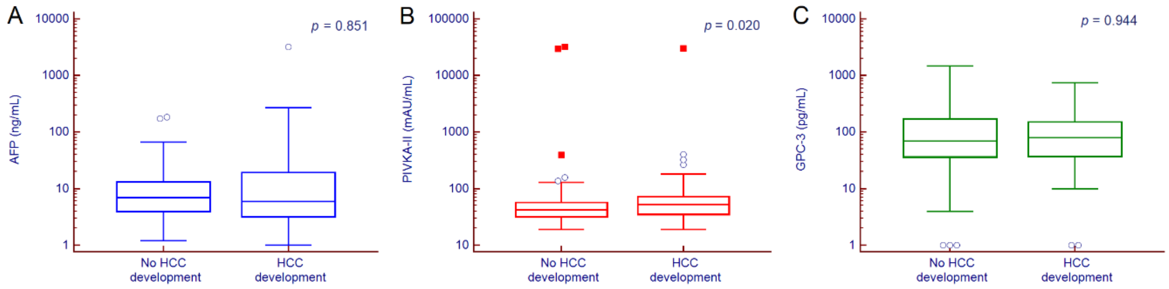

Figure S1. AFP (**A**), PIVKA-II (**B**) and GPC-3 (**C**) serum values in patients with cirrhosis that developed or not HCC during 36 months of follow-up. Hollow circles indicate values that are smaller than the lower quartile minus 1.5 times the interquartile range, or larger than the upper quartile plus 1.5 times the interquartile range; red squares indicate values that are smaller than the lower quartile minus 3 times the interquartile range, or larger than the upper quartile plus 3 times the interquartile range. Abbreviations–alpha-fetoprotein (AFP), hepatocellular carcinoma (HCC), glypican-3 (GPC-3), protein induced by vitamin K absence or antagonist II (PIVKA-II).

© 2020 by the authors. Licensee MDPI, Basel, Switzerland. This article is an open access article distributed under the terms and conditions of the Creative Commons Attribution (CC BY) license (http://creativecommons.org/licenses/by/4.0/).
